# Mass Administration of Ivermectin for the Elimination of Onchocerciasis Significantly Reduced and Maintained Low the Prevalence of *Strongyloides stercoralis* in Esmeraldas, Ecuador

**DOI:** 10.1371/journal.pntd.0004150

**Published:** 2015-11-05

**Authors:** Mariella Anselmi, Dora Buonfrate, Angel Guevara Espinoza, Rosanna Prandi, Monica Marquez, Maria Gobbo, Antonio Montresor, Marco Albonico, Marcia Racines Orbe, Juan Martin Moreira, Zeno Bisoffi

**Affiliations:** 1 Centro de Epidemiología Comunitaria y Medicina Tropical (CECOMET), Esmeraldas, Ecuador; 2 Centro per le Malattie tropicali, Negrar (Verona), Italy; 3 Facultad de Ciencias Medicas, Universidad Central del Ecuador, Quito, Ecuador; 4 Department of Control of Neglected Tropical Diseases (NTD), World Health Organization (WHO), Geneva, Switzerland; 5 Fondazione Ivo de Carneri, Milano, Italy; 6 Centro per le Malattie tropicali, Ospedale Sacro Cuore, Negrar (Verona), Italy; James Cook University, AUSTRALIA

## Abstract

**Objectives:**

To evaluate the effect of ivermectin mass drug administration on strongyloidiasis and other soil transmitted helminthiases.

**Methods:**

We conducted a retrospective analysis of data collected in Esmeraldas (Ecuador) during surveys conducted in areas where ivermectin was annually administered to the entire population for the control of onchocerciasis.

Data from 5 surveys, conducted between 1990 (before the start of the distribution of ivermectin) and 2013 (six years after the interruption of the intervention) were analyzed. The surveys also comprised areas where ivermectin was not distributed because onchocerciasis was not endemic.

Different laboratory techniques were used in the different surveys (direct fecal smear, formol-ether concentration, IFAT and IVD ELISA for *Strongyloides stercoralis*).

**Results:**

In the areas where ivermectin was distributed the strongyloidiasis prevalence fell from 6.8% in 1990 to zero in 1996 and 1999. In 2013 prevalence in children was zero with stool examination and 1.3% with serology, in adult 0.7% and 2.7%.

In areas not covered by ivermectin distribution the prevalence was 23.5% and 16.1% in 1996 and 1999, respectively. In 2013 the prevalence was 0.6% with fecal exam and 9.3% with serology in children and 2.3% and 17.9% in adults.

Regarding other soil transmitted helminthiases: in areas where ivermectin was distributed the prevalence of *T*. *trichiura* was significantly reduced, while *A*. *lumbricoides* and hookworms were seemingly unaffected.

**Conclusions:**

Periodic mass distribution of ivermectin had a significant impact on the prevalence of strongyloidiasis, less on trichuriasis and apparently no effect on ascariasis and hookworm infections.

## Author Summary

*Strongyloides stercoralis* (Ss) is a soil-transmitted helminth (STH) that is not yet targeted by control programs, although it is highly prevalent in many areas of the world and may cause severe consequences, in particular to immunosuppressed patients, with a high fatality rate. Unfortunately, albendazole, the drug most commonly used for the control of the other STH (hookworm, *Ascaris lumbricoides* and *Trichuris trichiura*) has little effect on Ss. The drug of choice, ivermectin, has been extensively used in mass drug administration (MDA) for the filarial worms *Onchocerca volvulus* and *Wuchereria bancrofti*. In the province of Esmeraldas, in Ecuador, we studied Ss (and other STH) prevalence from 1990 (prior to MDA initiation) to 2013 (6 years after MDA cessation) in rural communities where MDA was regularly executed for onchocerciasis compared with neighboring communities where ivermectin was not distributed because onchocerciasis was not present. Ss prevalence remained high over the years in the areas with no MDA, while in those with MDA prevalence fell to zero, and remained very low 6 years after MDA cessation. A less important effect was observed for *T*. *trichiura*. Adding ivermectin to MDA programs for STH would importantly contribute to the control of Ss infection.

## Introduction

The Onchocerciasis Elimination Programme of the Americas (OEPA) is a regional initiative with the goal of eliminating morbidity and interrupting transmission of river blindness in six endemic countries in the Americas [1]. In Ecuador, the main endemic focus of onchocerciasis was in the province of Esmeraldas, North of the country, where the elimination of onchocerciasis was achieved thanks to a community-based, sustained effort, based on the mass drug administration (MDA) of ivermectin once a year from 1991 to 2000, then twice, from 2001 to 2007 in all communities along Santiago river where onchocerciasis was endemic (while in the other endemic areas the treatment was carried on until 2009). Ivermectin was administered to adults of any age and to chidren over 5 years. A high level of coverage (ranging from 81.9% to 98.0% at each treatment instance) was obtained until 1996 [2], to increase in the following years to >90% in most treatment sessions and until the end of the program [3].

There is no doubt that ivermectin is also effective on *Strongyloides stercoralis* [4] and community-wide preventive chemotherapy has been proposed [5]. This paper is the first published evidence to our knowledge that such a strategy would be successful. Objective of this work is to estimate the effect of ivermectin MDA on the prevalence of *S*. *stercoralis* as well as of other soil transmitted helminths (STH) (*Ascaris lumbricoides*, *Trichuris trichiura* and hookworm) through: a) comparison of results from fecal surveys carried out before, during and after the distribution program; b) comparison of results from fecal and serological surveys carried out in areas targeted versus areas not targeted to ivermectin treatment, during and after the onchocerciasis elimination program.

## Material and Methods

### Study areas

**Fig 1:**
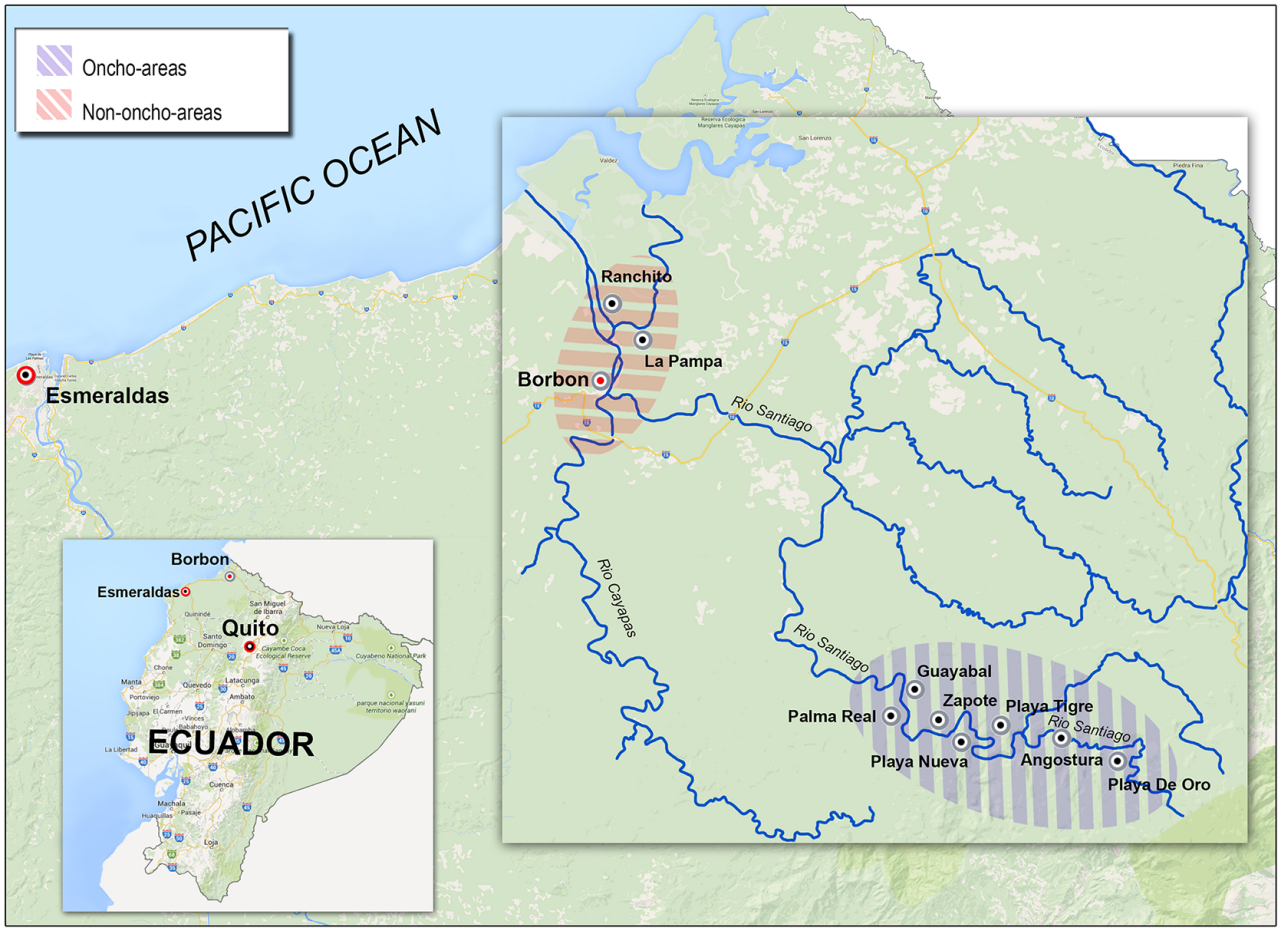
Map indicating the areas submitted and not submitted to ivermectin MDA for onchocerciasis (Oncho-areas and Non-oncho-areas, respectively).

The study area (Fig 1) is Borbon district, a rural tropical forest area located in the basin of the Cayapas, Santiago and Onzole rivers, bordering at a longitude of 78°30’ and 79°05’ West and a latitude 1°12’ and 0°35’, North, in its extreme limits, and at an altitude at sea level under 40 m.

The zone is inhabited by Afro-descendant people and the indigenous Chachi people, although there is a segment of mestizo population linked to a process of land colonization from other provinces of Ecuador.

The main economic activities are agriculture, fishing, and exploitation of forest resources. According to the data of the System of Social Indicators of Ecuador, in this area, 94.5% of the population are under the poverty line and 52.7% is in extreme poverty, currently only 16.36% of families have access to piped water at homes, 59.9% have access to latrines but only 3.6% are connected to sewage.

### Surveys

Five, cross-sectional surveys were carried out by CECOMET—Esmeraldas, Universidad Nacional of Quito and CTD—Negrar, Italy, with the field collaboration of the local health district Director and personnel, from 1990 to 2013, in areas along Santiago river where ivermectin was distributed for onchocerciasis elimination (Oncho-areas) and in areas around the village of Borbon where ivermectin was not distributed as they were not affected by onchocerciasis (Non-oncho-areas) (Fig 1).

1. In January, 1990, a first survey was carried out in adult population (N = 118) living in 7 communities along Santiago River, all of them to be then included (in April, same year) in the MDA program of ivermectin. The purpose of this survey was to obtain baseline data on intestinal parasites prior to ivermectin administration. Adults living in the study area were selected through convenience sampling. All those who had accepted to be regularly skin-snipped in a pilot project for onchocerciasis mass treatment were also submitted to a single stool examination. The laboratory method used was microscopic examination of direct fecal smear at the “Laboratorio de investigaciones clinicas” of Voz Andes Hospital in Quito.
2. In 1996, during the mass administration program for onchocerciasis, a follow-up survey on adult population (N = 112) was carried out in the same communities. The laboratory method used was microscopic examination of formol-ether concentrated stool at CTD.
3. In 1996 a fecal survey was also performed on school children (age 5–17 years), in the context of school health program, both in the 7 treated communities (N = 113) and in the area without ivermectin administration (N = 200): the village of Borbon and two close communities, an area outside the onchocercosis endemic focus and therefore not treated (Fig 1). All school children were offered to be tested.

The laboratory method used was microscopic examination of formol-ether concentrated stool at CTD; serology for *S*. *stercoralis* with Immune Fluorescence Antibody Test (IFAT) was also performed at CTD (in-house method described in detail elsewhere) [6]. IFAT cutoff titer for certain cases was >80 (the test is virtually 100% specific at this titer) [6]. Final result was defined as certain in case of positive stools AND/OR serology above the predetermined cutoff (1/80), possible in case of negative stools and positive serology (but below the cutoff), and negative in case of negative stools and serology. For logistical reasons, serology was only performed in children of the untreated area.

1. In 1999, a fecal survey was performed again on school children (age 5–17 y) in both areas (N = 102 in Oncho-areas and 161 in Non-oncho-areas). The laboratory method used was microscopic examination of formol-ether concentrated stool at CTD.
2. In 2013, six years after cessation of ivermectin administration, in the same areas where the previous surveys were carried out, a new, cross-sectional survey was performed, including both children (age 5–17 y, N = 275 in Oncho-areas and 183 in Non-oncho-areas) and adults (N = 150 in Oncho-areas and 156 in Non-oncho-areas). Testing was offered to all children’s parents.

Laboratory methods used were: microscopic examination of formol-ether concentrated stool at CTD; serology for *S*. *stercoralis* with IVD-ELISA (S-stercoralis serology Microwell ELISA Kit, IVD Research Carlsbad, CA) was carried out at the reference laboratory for parasitology of Universidad Nacional of Quito. The method is described in detail elsewhere [6]. Cutoff for certain cases was ≥0.5 Optical Density (the test is virtually 100% specific at this OD) [6]. Final results were defined as certain, possible or negative according to fecal and serologic results, similarly to 1996.

**Fig 2:**
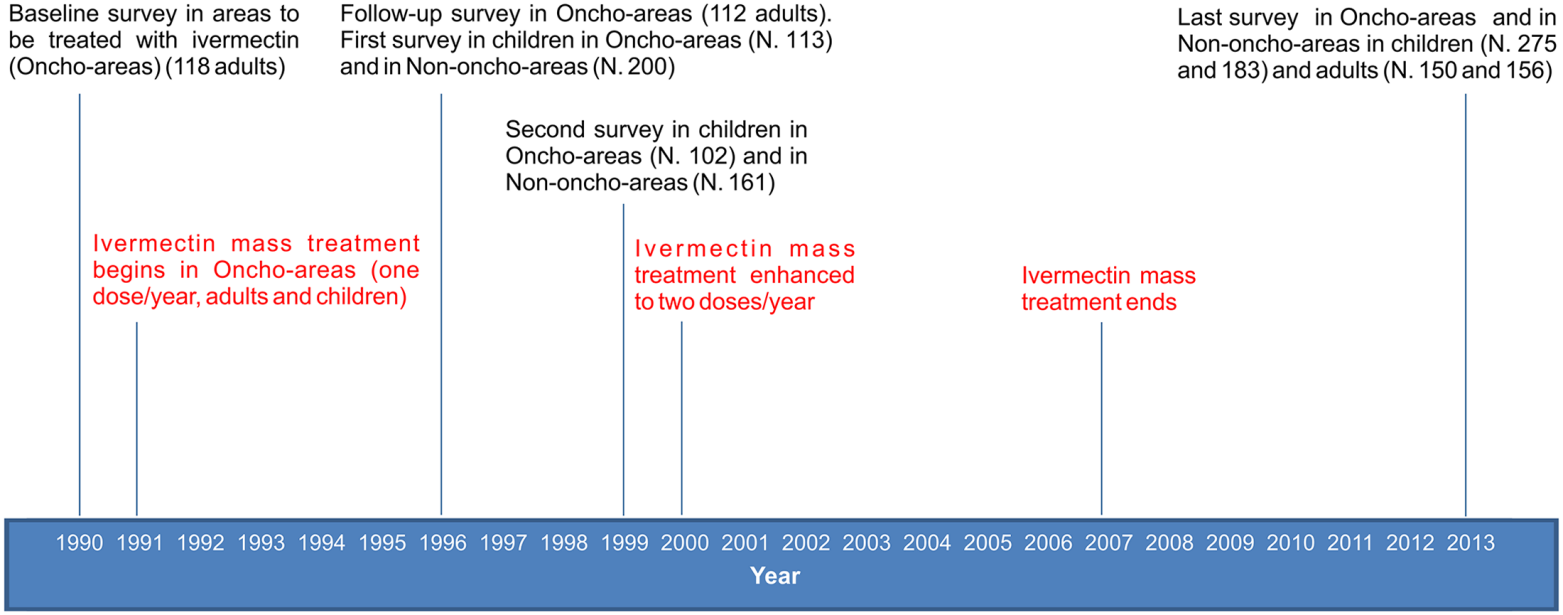
Timeline of the prevalence surveys and of ivermectin mass administration.

The timeline of the different surveys and of ivermectin MDA is reported in Fig 2.

In all the surveys the laboratory personnel received pre coded, anonymous samples and were unaware of any characteristics of the subjects, including their origin.

### Data entry and analysis

Data was colleted in Excel and analyzed using the software STATA IC 14 (StataCorp, 4905 Lakeway, College Station, TX, 77845, USA—www.stata.com).

All variables considered were categorical. The absolute, relative and (when indicated) cumulative frequencies were calculated with the respective 95% Confidence Intervals (CI).

### Ethical issues

Informed consent was obtained from members of the communities and from the children’s parents or guardians, before the collection of any biological samples. Consent was obtained verbally for surveys carried out from 1990 to 1999, that were not submitted to the Ethics Committee, as they were not primarily intended as research, but rather as screening and treatment programs promoted by the Ministry of Health, including School Health program. All exams were carried out free of charge, results were made available to all study subjects and treatment was also offered free of charge, when indicated, and according to the best local practice guidelines. The Ethics Committee of the Central University of Ecuador in Quito (“Comité de Bioetica”—COBI) approved the study protocol in December, 2012, including the last survey (for which a written informed consent was obtained from all the study subjects and/or their guardians) and the retrospective analysis of the previous ones. A minor amendment was approved in November, 2013.

## Results

### S. stercoralis

Longitudinal data in adults in Oncho-areas from 1990 to 1996 showed that prevalence fell from 6.8%; (C.I. 3.5–12.8) in 1990 (just before the start of MDA with ivermectin) to 0 (C.I. 0.0–3.3) in 1996 (Table 1).

**Table 1:** Adults, 1990 (direct stool examination) vs 1996 (formol—ether concentration) in Oncho-areas.

STH	Year 1990 (118 adults)	%	95% CI	Year 1996 (112 adults)	%	95% CI
***S*. *stercoralis***	**8**	**6.8%**	**3.5–12.8**	**0**	**0%**	**0.0–3.3**
*A*.*lumbricoides*	78	66.1%	57.2–74.0	68	60.7%	51.5–69.3
Hookworm	20	16.9%	11.3–24.7	30	26.8%	19.5–35.7
*T*. *trichiura*	22	18.6%	12.6–26.6	24	21.4%	14.9–29.9

In children in 1996, the prevalence evaluated with formol-ether concentration was 0 (CI 0.0–3.3) in Oncho-areas and 23.5% (C.I. 18.2–29.8) in Non-oncho-areas (Table 2).

**Table 2:** Children (5–17 years), 1996, Oncho-areas versus Non-oncho-areas (formol–ether concentration).

STH	Oncho-areas (113)	%	95% CI	Non-oncho-areas (200)	%	95% CI
***S*. *stercoralis***	**0**	**0%**	**0–3.3**	**47**	**23.5%**	**18.2–29.8**
*A*.*lumbricoides*	77	68.1%	59.1–76.0	146	73%	66.5–78.7
Hookworm	44	38.9%	30.5–48.2	39	19.5%	14.6–25.5
*T*. *trichiura*	32	28.3%	20.8–37.2	172	86%	80.5–90.1

In the same year, serology (IFAT) for *S*. *stercoralis* was carried out in Non-oncho-areas only. Results at different cutoff are reported in Table 3.

**Table 3:** 1996–Prevalence of anti–*S*. *stercoralis* antibodies (IFAT), Non-oncho-areas (200 children 5–17 y).

IFAT titer	Freq	%	Cumulative
**0**	68	34,0%	34,0%
**20**	37	18,5%	52,5%
**40**	25	12,5%	65,0%
**80**	22	11,0%	76,0%
**160**	16	8,0%	84,0%
**320**	20	10,0%	94,0%
**640**	8	4,0%	98,0%
**1280**	4	2,0%	100,0%
**Total**	200	100,0%	100,0%

The results were further classified according to the combined result of fecal examination and IFAT as defined in Materials and Methods. Prevalence was estimated between 35.5% (C:I: 29.2–42.4) considering certain cases and 67.5% (C.I: 60.7–73.6) if we included also possible cases (Table 4).

**Table 4:** 1996–Classification of Ss infection according to the combined result of fecal examination plus serology (IFAT), Non-oncho-areas (200 children 5–17 y).

Composite reference standard result	Freq	%	95% CI
Negative (negative stool and IFAT = 0)	65	32.5%	26.4–39.3
Possible (negative stool and IFAT = 20 to 80)	64	32.0%	25.9–38.8
Certain (positive stool or IFAT > 80)	71	35.5%	29.2–42.4

Results from cross-sectional studies in children, year 1999, Oncho-areas versus Non-oncho-areas are shown in Table 5. In Oncho-areas, *S*. *stercoralis* prevalence in children evaluated with formol-ether concentration was 0 (C.I. 0.0–3.6), while in Non-oncho-areas the prevalence was 16.1% (C.I. 10.8–22.8).

**Table 5:** Year 1999 (formol—ether concentration), children 1–17 y.

STH	Oncho-areas (102)	%	95% CI	Non-oncho-areas (161)	%	95% CI
***S*. *stercoralis***	**0**	**0%**	**0–3.6**	**26**	**16.1%**	**10.8–22.8**
*A*.*lumbricoides*	57	55.9%	46.2–65.1	83	51.6%	43.6–59.5
Hookworm	46	45.1%	35.8–54.8	27	16.8%	11.4–23.5
*T*. *trichiura*	46	45.1%	35.8–54.8	114	70.8%	63.1–77.7

In 2013, six years after the conclusion of mass treatment program, and 14 years after the last survey, the prevalence of *S*. *stercoralis* measured using stool examination in children in Oncho-areas was 0% (C.I. 0.0–1.4) (Table 6).

**Table 6:** Year 2013 (formol–ether concentration): Children and adults.

STH	Oncho-areas (275 children)	% (CI)	Non-oncho-areas (183 children)	% (CI)	Oncho-areas (150 adults)	% (CI)	Non-oncho-areas (156 adults)	% (CI)
***S*. *stercoralis***	**0**	**0 (0–1.4)**	**1**	**0.6 (0.1–3.0)**	**1**	**0.7 (0.1–3.7)**	**4**	**2.3 (1.0–6.4)**
*A*.*lumbricoides*	**130**	**47.3 (41.5–53.2)**	**97**	53.0 (45.8–60.1)	56	37.3 (30.0–45.3)	**63**	40.4 (33.0–8.2)
Hookworm	**24**	**8.7 (5.9–12.7)**	**10**	5.5 (3.0–9.8)	7	4.7 (2.3–9.3)	**3**	1.9 (0.7–5.5)
*T*. *trichiura*	**26**	**9.5 (6.5–13.5)**	**70**	38.3 (31.5–45.5)	5	3.3 (1.4–7.6)	**28**	18.0 (12.7–24.7)

By serology, the prevalence was 1.1% (C.I. 0.4–3.2) and 4.0% (C.I. 2.3–7.0) for certain and certain plus possible cases, respectively (Table 7).

**Table 7:** Year 2013: Classification of Ss infection according to the combined result of fecal examination plus serology, by area and age group.

Ss infection	Oncho-areas (275 children 1–17 y)	Non-oncho-areas (183 children 1–17 y)	Oncho-areas (150 adults)	Non-oncho-areas (156 adults)
Not infected	264 (96.0%)	153 (83.6%)	137 (91.3%)	97 (62.2%)
Possible	8 (2.9%)	13 (7.1%)	9 (6.0%)	31 (19.9%)
Certain	3 (1.1%)	17 (9.3%)	5 (3.3%)	28 (17.9%)

In Non-oncho-areas, the prevalence in children resulted 0.6% (CI 0.1–3.0) at stool examination (Table 6), while combining results of fecal examination and serology the prevalence was 9.3% (C.I. 5.9–14.4) and 16.4% (C.I. 11.8–22.4) for certain and certain plus possible cases, respectively (Table 7). In adults, *S*. *stercoralis* prevalence measured with stool examination was 0.7% (CI 0.1–3.7) in Oncho-areas and 2.3% (CI 1.0–6.4) in Non-oncho-areas (Table 6). By serology, in Oncho-areas the prevalence of certain and certain plus possible cases was 2.7% (C.I. 0.7–6.7) and 9.3% (C.I. 5.6–15.1), respectively, while in Non-oncho-areas the prevalence was 17.9% (C.I. 12.7–24.7) and 37.8% (C.I. 30.6–45.6), respectively (Table 7).

### Other STH

The prevalence of *A*. *lumbricoides*, *T*. *trichiura* and hookworm is summarized in Tables 1, 2, 5 and 6. In adults the prevalence of the three STH in 1990, before the start of ivermectin mass treatment, and in 1996 was similar (66.1 vs 60.7%, 16.9 vs 26.8% and 18.6 vs 21.4%, respectively).

In children, comparing the two areas during the mass administration program in 1996 and in 1999 (Tables 2 and 5), the prevalence of *A*. *lumbricoides* was similar in both areas (68.1 vs 73% and 55.9 vs 51.6%, NS), while that of hookworm was significantly higher in Oncho-areas: 38.9% (CI 30.5–48.2) vs 19.5% (CI 14.6–25.5) in 1996 and 45.1% (CI 35.8–54.8) vs 16.8% (CI 11.4–23.5) in 1999. Conversely, prevalence of *T*. *trichiura* was significantly lower in Oncho-areas: in 1996, 28.3% (CI 20.8–37.2) vs. 86% (CI 80.5–90.1), and in 1999, 45.1% (CI 35.8–54.8) vs 70.8% (CI 63.1–77.7).

In 2013 the prevalence of the other STH declined in both areas and age groups, with the partial exception of *A*. *lumbricoides*. Prevalence of the latter in children was 47.3% (CI 41.5–53.2) in Oncho-areas and 53.0% (CI 45.8–60.1) in Non-oncho-areas, in adults it was 37.3 (CI 30.0–45.3) and 40.4 (CI 33.0–48.2), respectively. Hookworm prevalence declined in both areas and age groups, but remained higher in Oncho-areas: 8.7 (CI 5.9–12.7) vs. 5.5 (CI 3.0–9.8) in children, and 4.7 (CI 2.3–9.3) vs. 1.9 (CI 0.7–5.5) in adults, though the difference was not statistically significant. Conversely, *T*. *trichiura* prevalence remained lower in Oncho-areas: 9.5% (CI 6.5–13.5)vs 38.3% (CI 31.5–45.5) in children and 3.3% (CI 1.4–7.6) vs 18.0% (CI 12.7–24.7) in adults.

## Discussion

The results of several surveys reported in this unique study document the evolution of the prevalence of selected STH over a quarter of a century, starting in 1990, prior to the onset of the MDA with ivermectin, and up to 2013, six years after the conclusion of the mass administration program.

The mass administration (once a year from 1991 to 2000, then twice a year until 2007) of this drug in the context of onchocerciasis control has caused a significant and sustained impact on the prevalence of *S*. *stercoralis* infection. The study was conducted in areas with very high prevalence of strongyloidiasis. The benefits of the MDA were still present in 2013, six years after the conclusion of ivermectin distribution. Over time the prevalence of *S*. *stercoralis* measured by microscopy declined in both the age groups considered in Non-oncho-areas (remaining virtually absent in Oncho-areas), while serology still indicated a higher prevalence in Non-oncho-areas than in Oncho-areas. The difference between the results of serology and those of microscopy was more marked than in 1996 survey.

We hypothesized that this might be due to the higher sensitivity of serology because: i) we recently showed that serology is virtually 100% specific above a given cutoff [6] ii) the serologic titer sharply declines in a matter of months after effective treatment [7–11], and ivermectin has not been available up to now in Non-oncho-areas. Moreover, the striking difference in prevalence between the two areas would not to be explained by problems inherent to the methods used.

The higher sensitivity of serology is therefore the most plausible explanation as documented by many studies [9,12–18]. It is possible that in 2013 the average larval load in feces was lower, partly because of the widespread (albeit irregular) use of albendazole since the year 2000 (Mariella Anselmi, personal communication). This, along with an improvement in hygienic conditions and housing in the area of Borbon in the past few years, has seemingly had an important effect on other STH (see below). Albendazole, however, is poorly effective on *S*. *stercoralis* [19], and the repeated administration of this drug could have reduced the parasitic burden in most infected subjects below a detection threshold for the (far from ideal) fecal methods used, but not for the much more sensitive serology [20].

It is noteworthy that, six years after the administration of ivermectin in Oncho-areas, the prevalence of *S*. *stercoralis*, measured with serological methods, remained much lower than elsewhere. However, there are some adults who resulted positive despite several courses of ivermectin. This might be due to the persistence of the transmission in the area (hypothesis that might also be confirmed by the fact that there are younger children infected, although they might also have acquired strongyloidiasis moving to other areas). HTLV1, that is present in Esmeraldas province [21], may have played a role in the lack of response to repeated ivermectin courses in a few subjects, posing obstacles to the definitive eradication of *S*. *stercoralis*. Ad hoc surveys would be necessary to confirm this hypothesis. This issue also raises the relevant question of which markers of cure should be used for *S*. *stercoralis* infection in endemic areas.

Regarding other STH, the prevalence of *A*. *lumbricoides*, hookworm and *T*. *trichiura* was high or very high before and during the MDA program. However, while in 1996 and in 1999 the prevalence of *A*. *lumbricoides* and hookworm appeared to be unaffected by ivermectin administration, *T*. *trichiura* prevalence was, conversely, significantly lower in Oncho-areas, suggesting an effect of ivermectin MDA on the prevalence of this parasite. This coincides with similar results found by other researchers [22], although in other studies ivermectin has been shown to have a good effect on ascariasis [23–26]. A general improvement in hygiene and sanitation conditions over the years has certainly played a role in the reduction of STH prevalence.

This study suffers from important limitations: 1) the difference in the diagnostic methods used, some of them far from ideal for the diagnosis of *S*. *stercoralis*, the main target of our study; 2) the sampling methodology that was not on a random basis but rather based on convenience criteria, although the number of the subjects tested in each community represented an important proportion of the whole population; 3) the very long time interval elapsed between the surveys carried out during and after ivermectin intervention, thus making it difficult to closely follow the evolution of prevalence over time; 4) the use of *S*. *stercoralis* serology, the most sensitive diagnostic method for this parasite, was only possible in 1996 during the ivermectin program, and only in Non-oncho-areas. Only in the last survey (2013), 6 years after the conclusion of the intervention, was serology performed in both areas; 5) the diagnostic methods used did not allow to quantify the intensity of infection for the other STH, therefore the impact of ivermectin MDA on the latter might be underestimated. While duly acknowledging these limitations, mostly inherent to the retrospective study design, we believe that the effect of ivermectin on the prevalence of *S*. *stercoralis* is apparent, and that the difference between the two areas is so huge that it is unlikely to be affected by any bias.

### Conclusions

In an area with high prevalence of strongyloidiasis, regular ivermectin mass administration has showed an important impact on the prevalence of this parasite. This finding, obtained through a retrospective study, needs to be confirmed by appropriate longitudinal studies, including randomized community trials, however it clearly indicate the possibility of controlling strongyloidiasis with large scale distribution of ivermectin.

In addition the data from our study also support the possibility of routinely include ivermectin during preventive chemotherapy campaigns targeting soil transmitted helminthiasis because of the beneficial effect on trichuriasis, a parasite that is more difficult to treat with albendazole and mebendazole only [27,28], and the possible advantage of the albendazole—ivermectin combination therapy to prevent the insurgence of drug resistance [29]. Moreover it could be also considered to replace with the latter combination the commonly-used regimen of albendazole and diethylcarbamazine for preventative community treatment (PCT) of lymphatic filariasis where *S*. *stercoralis* and LF are co-endemic. And finally, PCT with ivermectin has also been proposed to control scabies [30], which would be an important added value.

## Supporting Information

S1 Database Study database. (XLS)
